# Cinacalcet studies in pediatric subjects with secondary hyperparathyroidism receiving dialysis

**DOI:** 10.1007/s00467-020-04516-4

**Published:** 2020-05-04

**Authors:** Bradley A. Warady, Eric Ng, Laura Bloss, May Mo, Franz Schaefer, Justine Bacchetta

**Affiliations:** 1 Division of Pediatric Nephrology, Children’s Mercy Kansas City, Kansas City, MO USA; 2 Amgen Inc., Thousand Oaks, CA USA; 3 Heidelberg University Hospital, Heidelberg, Germany; 4 Department of Pediatric Nephrology, Rheumatology and Dermatology, Femme Mère Enfant Hospital, Bron, France

**Keywords:** Cinacalcet, CKD-MBD, Dialysis, Pediatric, sHPT

## Abstract

**Background:**

Secondary hyperparathyroidism (sHPT), a complication of chronic kidney disease (CKD) characterized by persistently elevated parathyroid hormone (PTH), alterations in calcium-phosphorus homeostasis, and vitamin D metabolism, affects 50% of children receiving dialysis. A significant proportion of these children develop CKD-mineral and bone disorder (CKD-MBD), associated with an increased risk of fractures and vascular calcification. The standard of care for sHPT in children includes vitamin D sterols, calcium supplementation, and phosphate binders. Several agents are approved for sHPT treatment in adults undergoing dialysis, including vitamin D analogs and calcimimetics, with limited information on their safety and efficacy in children. The calcimimetic cinacalcet is approved for use in adults with sHPT on dialysis, but is not approved for pediatric use outside Europe.

**Methods:**

This review provides dosing, safety, and efficacy information from Amgen-sponsored cinacalcet pediatric trials and data from non-Amgen sponsored clinical studies.

**Results:**

The Amgen cinacalcet pediatric clinical development program consisted of two Phase 3 randomized studies, one Phase 3 single arm extension study, one open-label Phase 2 study, and two open-label Phase 1 studies. Effects of cinacalcet on PTH varied across studies. Overall, 7.4 to 57.1% of subjects who received cinacalcet in an Amgen clinical trial attained PTH levels within recommended target ranges and 22.2 to 70.6% observed a ≥ 30% reduction in PTH. In addition, significant reductions in PTH were demonstrated in all non-Amgen-supported studies.

**Conclusions:**

To help inform the pediatric nephrology community, this manuscript contains the most comprehensive review of cinacalcet usage in pediatric CKD patients to date.

**Electronic supplementary material:**

The online version of this article (10.1007/s00467-020-04516-4) contains supplementary material, which is available to authorized users.

## Introduction

End-stage renal disease (ESRD) in children is rare with 18–100 per million patients requiring renal replacement therapy globally [1]. Children are priority candidates for kidney transplantation; as a result, the point prevalence of pediatric patients on dialysis is relatively low (e.g., < 1% [3,151]) of all US patients on dialysis (466,607) [2].

Secondary hyperparathyroidism (sHPT) is a compensatory complication of chronic kidney disease (CKD). sHPT is characterized by persistently elevated parathyroid hormone (PTH) concentrations in serum or plasma, and it represents an adaptive response that serves primarily to maintain calcium homeostasis systemically as kidney function declines [3]. Early alterations in fibroblast growth factor 23 (FGF23), vitamin D metabolism, and calcium and phosphorus regulation lead to a reduction in signaling through the calcium-sensing receptor (CaSR) and an increase in PTH secretion, resulting in higher PTH concentrations. Late in the course of CKD, phosphorus retention and overt hyperphosphatemia, together with skeletal resistance to the calcemic action of PTH, can also affect calcium metabolism adversely and further increase PTH secretion among patients with more advanced CKD [3–6]. Collectively, these changes in calcium and phosphorus concentrations and vitamin D metabolism contribute to the progression of sHPT, which generally worsens in severity over time if left untreated. sHPT affects 50% of children receiving dialysis [7].

The consequences of sHPT include the development of CKD-mineral bone disorder (CKD-MBD), defined as a systemic disorder of mineral and bone metabolism due to CKD that is manifested by either one or a combination of the following: (1) abnormalities in calcium, phosphorus, PTH, or vitamin D metabolism; (2) abnormalities in bone turnover, mineralization, volume, linear growth, or strength; and (3) vascular or other soft tissue calcification [8]. In children, CKD-MBD develops early during CKD, such that 50% of children with stage 3 CKD, and 60% with stage 4/5 CKD have manifestations of CKD-MBD [9]. Moreover, these patients can experience either calcium deficiency, potentially causing impaired mineralization and an increased fracture risk [9–11], or calcium excess, linked with vascular calcification [7].

The recommended standard of care (SOC) for the treatment of sHPT includes the use of vitamin D sterols, calcium supplementation, and phosphate binders (calcium-based and non-calcium-based, with the exception of aluminum salts that are contraindicated) [12]. Current pediatric consensus guidelines recommend that the SOC for pediatric patients should specifically focus on maintaining serum calcium and phosphate within the age-appropriate normal range [12–14]. Additionally, the National Kidney Foundation Kidney Disease Outcomes Quality Initiative (NKF K/DOQI) recommends PTH levels within a target range (> 150 to 300 pg/mL) [15], while the European Pediatric Dialysis Working Group (EPDWG) has recommended that PTH be monitored monthly and kept at two to three times the upper limit of the normal range in advanced CKD [12].

Several agents have been approved by the US Food and Drug Administration (FDA) and the European Medicines Agency (EMA) for the treatment of sHPT in adult patients undergoing dialysis. These include the vitamin D analogs doxercalciferol, paricalcitol, calcitriol, and the calcimimetics cinacalcet and etelcalcetide. Despite the availability of these therapies for adults, there is limited information on the safety and efficacy of these products in children, and pediatric formulations are generally not commercially available. Treatment with vitamin D sterols may not completely control sHPT in children with CKD, either due to insufficient efficacy or limited use resulting from the subsequent development of hypercalcemia and hyperphosphatemia. Furthermore, different therapeutic approaches to sHPT have differential effects on FGF23. Vitamin D tends to increase FGF23, and cinacalcet tends to lower FGF23, while the effects of calcium and non-calcium-based phosphate binders are variable [16–18].

The calcimimetic cinacalcet is an allosteric modulator of the CaSR, increasing receptor sensitivity to calcium and thereby increasing the potency of extracellular calcium resulting in decreased PTH secretion and production [19–21]. Cinacalcet has been approved by the FDA and EMA for use in adults with sHPT on dialysis [20]. In the EU, cinacalcet is approved for patients aged 3 years and older with ESRD on maintenance dialysis therapy in whom sHPT is not adequately controlled with SOC therapy [22]. To help address the unmet clinical need for additional therapies to treat sHPT in children, Amgen conducted an extensive pediatric development program for cinacalcet to investigate the dosing, safety, and efficacy in pediatric patients with sHPT on dialysis. Overall, 142 children were enrolled in interventional clinical trials in the Amgen-sponsored pediatric clinical development program; 103 of whom received cinacalcet. An additional 113 children received cinacalcet in observational studies that were part of the cinacalcet pediatric development program. Finally, safety and efficacy data are available from 60 children who received cinacalcet in five non-Amgen-supported clinical studies [23–27]. Herein, we provide a comprehensive review of all cinacalcet clinical studies in pediatric subjects to help inform the pediatric nephrology community.

## Methods

After establishing cinacalcet bioavailability and bioequivalence of a pediatric formulation (Study 20070293), Amgen undertook a series of clinical trials. The Amgen cinacalcet pediatric clinical development program consisted of two phase 3 randomized studies, one phase 3 single arm extension study, one open-label phase 2 study, and two open-label phase 1 studies**.** Study details, including study objective, dosing regimen, and study populations, are provided in Table 1. In all multidose studies, the cinacalcet starting dose was titrated based on PTH and calcium thresholds, and safety assessments. Additional real-world observational data were available from a multicenter, retrospective, chart review study and a prospective cohort registry study. To summarize data outside of Amgen-supported studies, we undertook a comprehensive PubMed literature review (through 03 January 2019) of studies evaluating the use of cinacalcet in pediatric subjects on dialysis (Table 2). Preclinical studies, case reports, and review articles were omitted from this manuscript. Studies were also excluded if subjects had primary or tertiary hyperparathyroidism, hyperparathyroidism due to CaSR mutations, parathyroid carcinoma or malignancy, were not on dialysis, or had CKD 4 or lower. All Amgen study protocols, subject information, and informed consent forms were reviewed and approved by the IRB/IEC for each study center. All Amgen studies were conducted in accordance with applicable country regulations and International Conference on Harmonisation (ICH) Good Clinical Practice (GCP) regulations/guidelines.

**Table 1:** Amgen-supported studies of cinacalcet in pediatric subjects

Study ID(s), type, and objectives	Dose regimen	Study population
Clinical trials		
^a^20070208/NCT01277510 Double-blind, placebo-controlled Phase 3 RCT Primary objective To assess the safety and tolerability of cinacalcet including evaluation of AEs and incidence of hypocalcemia Other objectives To evaluate efficacy and bioanalytical parameters	Starting dose*:* ≤ 0.2 mg/kg/day based on dry weight Titration: See Table 4 Provision: Either as capsules for sprinkling or tablets Planned treatment duration: • Double-blind: 30 weeks • Open-label: 30 weeks	*N* = 43 (*n* = 22 cinacalcet; *n* = 21 placebo) Key inclusion criteria • Aged 6 to < 18 years • Dry weight ≥ 12.5 kg at screening • PTH > 300 pg/mL • cCa ≥ 8.8 mg/dL • Receiving hemodialysis or peritoneal dialysis for ≥ 2 months before randomization Key exclusion criteria • PTx within 6 months before or anticipated within 6 months after randomization • Treatment with cinacalcet within 1 month before randomization • A new onset of seizure or worsening of a preexisting seizure disorder within the last 3 months • Scheduled date for kidney transplant from a known living donor that makes completion of the study unlikely
^b^20130356/NCT02138838 Open-label, active-controlled Phase 3 RCT Primary objective To assess the safety and tolerability of cinacalcet in pediatric subjects Other objectives To evaluate efficacy and bioanalytical parameters	Starting dose: ≤ 0.2 mg/kg/day (based on dry weight) Titration: See Table 4. Provision: Either as capsules for sprinkling or tablets Planned treatment duration: • Open-label: 20 weeks	*N* = 55 (*n* = 27* cinacalcet plus standard of care; *n* = 28 standard of care) *Only 25 of the enrolled patients received cinacalcet Key inclusion criteria • Aged 6 to < 18 years • PTH ≥ 300 pg/mL • cCa ≥ 8.8 mg/dL • Receiving hemodialysis or peritoneal dialysis for ≥ 30 days before screening Key exclusion criteria • History of congenital long QT syndrome, second or third-degree heart block, ventricular tachyarrhythmias, or other conditions associated with prolonged QT interval • Corrected QT interval (QTc) > 500 ms, using Bazett’s formula • QTc ≥ 450 to ≤ 500 ms, using Bazett’s formula, unless written permission to enroll is provided by the investigator after consultation with a pediatric cardiologist
^a, b^20110100/NCT01439867 Open-label, single-arm phase 2 study Primary objective To assess the safety and tolerability of cinacalcet in addition to standard of care in pediatric subjects age 28 days to < 6 years Other objectives Included PK, PD, and characterization of serum calcium	Starting dose: 0.25^a^ and 0.20^b^ mg/kg/day based on dry weight Titration: 1 mg to 60 mg Provision: 5 mg capsules for sprinkling or mixed with sucrose syrup for oral administration Planned treatment duration: • 26 weeks	*N* = 18 Key inclusion criteria • Age 28 days to < 6 years at enrollment^c^ • Dry weight ≥ 7 kg at the time of screening • Screening plasma PTH level > 300 pg/mL (31.8 pmol/L) from the central laboratory, and not received any cinacalcet therapy for at least 30 days prior to start of dosing • Screening cCa from the central laboratory: ○ ≥ 9.4 mg/dL (2.35 mmol/L) if age 28 days to < 2 years ○ ≥ 8.8 mg/dL (2.2 mmol/L) if age ≥ 2 to < 6 years • Serum phosphorus from the central laboratory: ○ ≥ 5.0 mg/dL (1.25 mmol/L) if age 28 days to < 1 year ○ ≥ 4.5 mg/dL (1.13 mmol/L) if age ≥ 1 to < 6 years • sHPT not due to vitamin D deficiency, per investigator assessment Key exclusion criteria • History of congenital long QT syndrome, second- or third-degree heart block, ventricular tachyarrhythmias or other conditions associated with prolonged QT interval • Corrected QT interval (QTc) > 500 ms, using Bazett’s formula • QTc ≥ 450 to ≤ 500 ms, using Bazett’s formula, unless written permission to enroll is provided by the investigator after consultation with a pediatric cardiologist
^b^20140159/NCT02341417 Open-label, Phase 3 extension study—extension of 20130356 and 20110100 Primary objective To characterize the long-term safety and tolerability of cinacalcet in pediatric subjects with CKD receiving dialysis Other objectives To assess change in PTH, corrected serum calcium, and phosphate	Starting dose: 0.2 mg/kg/day based on dry weight Titrations: • Subjects < 6 years old: 1, 2.5, 5, 7.5, 10, 15, 30, and 60 mg doses • Subjects ≥ 6 years old: 2.5, 5, 10, 15, 30, 60, 90, 120, and 180 mg doses Provision: 5-mg capsules for sprinkling or 30-mg film-coated tablets for swallowing Planned treatment duration: • 32 weeks	*N* = 28 (27 enrolled from Study 20130356; 1 enrolled from Study 20110100); 13/27 subjects were on SOC in the parent study and subsequently treated with cinacalcet during the extension study Key inclusion criteria • All subjects: ○ Dialysate calcium concentration ≥ 2.5 mEq/L at day 1 ○ More than 14 days between the last study visit in Study 20130356 or Study 20110100 and screening for Study 20140159 • All subjects from 20130356: ○ Completed treatment through week 20 or on study at the time of Study 20130356 termination ○ Dry weight ≥ 12.5 kg at day 1 of Study 20140159 • Subjects randomized to the 20130356 Standard of Care Arm only: ○ PTH ≥ 300 pg/mL (within 7 days of day 1) ○ cCa ≥ 8.8 mg/dL (within 7 days of day 1) • All subjects from 20110100: ○ Completed week 26 end of study visit or on study at the time of Study 20110100 termination ○ Dry weight ≥ 7 kg at day 1 of Study 20140159 Key exclusion criteria • Those listed above for Studies 20130356 and 20110100, plus: ○ Unstable chronic heart failure during screening ○ Received cinacalcet after the last study visit in Study 20130356 or Study 20110100 before day 1 of Study 20140159 ○ Scheduled date for kidney transplantation from a known living donor that makes completion of the study unlikely ○ Either new or recurrent cardiac ventricular arrhythmias requiring a change in treatment within 10 days prior to screening visit or day 1 of Study 20140159 screening ○ Hepatic impairment during screening
20090005/NCT01290029 Phase 1, open-label, single-dose study Primary objective To evaluate the safety and tolerability of a single-dose, oral administration of cinacalcet in pediatric subjects with CKD receiving dialysis Other objectives To evaluate the PK and PD of cinacalcet in pediatric subjects with CKD receiving dialysis	Starting dose: 0.25 mg/kg (2 h fasting pre- and postadministration) Titration: n/a Provision: 5 mg capsule Planned treatment duration: • Single dose	*N* = 14 Key inclusion criteria • Age 28 days to < 6 years • Body weight ≥ 6 kg at screening and at day − 1; gestational age 30 weeks; physical examination must be acceptable to investigator at screening and at day − 1 • Serum calcium within age-appropriate normal ranges per NKF-K/DOQI guidelines at screening and at day − 1 Key exclusion criteria • Current or historic malignancy • Cardiac ventricular arrhythmias within 28 days prior to screening • A gastrointestinal disorder or surgery • A new onset of seizure or worsening of a preexisting seizure disorder within 2 months prior to cinacalcet administration • Major surgery (involves general anesthesia or respiratory assistance) within 28 days prior to screening • History of prolongation of QT interval • Clinically significant abnormal electrocardiogram at screening and day 1
20030227 Phase 1, open-label, single-dose study. Primary objective To evaluate the safety and tolerability of a single-dose, oral administration of cinacalcet in pediatric subjects with CKD receiving dialysis Other objectives To evaluate the PK and PD of cinacalcet in pediatric subjects	Starting dose: 15 mg oral dose (12 h fasting) Titration: n/a Provision: Half of a 30-mg tablet Planned treatment duration: • Single dose, followed for 72 h after dosing	*N* = 12 Key inclusion criteria • Age 6 to < 18 years • Receiving dialysis for at least 1 month at time of screening and had not received any cinacalcet therapy for at least 2 weeks prior to day 1 dosing Key exclusion criteria • Not stated
Supportive real-world studies		
20090198 Retrospective chart review Primary objective To describe the safety and tolerability of cinacalcet; to describe changes in biochemical markers (PTH, calcium, and phosphate concentrations) Other objectives To describe method of administration, route, dose and frequency, use of other medications, and to describe bone markers	Starting dose: Various; median (min, max) 0.61 (0.1, 1.9) mg/kg/day Titration: Not stated Provision: Commercially available tablets crushed for administration orally or through nasogastric tube Planned follow-up • 1 month • 2 months • 3 months	*N* = 23 Key inclusion criteria • Diagnosed with CKD requiring maintenance dialysis • Diagnosed with sHPT • Treated with ≥ 1 dose of cinacalcet at any time prior to 31 August 2009 • Age < 6 years at the time cinacalcet was initiated Key exclusion criteria • Not stated
20120116 Multicenter prospective registry cohort study Primary objective To describe the prevalence of cinacalcet use among pediatric subjects receiving dialysis Other objectives To describe the characteristics of pediatric dialysis subjects who initiate cinacalcet and to describe the safety of cinacalcet use among these pediatric subjects receiving dialysis	Starting dose: Various; most frequent dose: 30 mg/day (43/80 subjects; 54%)^d^ Median weekly dose was 210 mg (range 21 to 840 mg) and the median (range) dose per kg of body weight per week was 4.8 mg/kg/week (1.1 to 32.3 mg/kg/week) Titration: Not stated Provision: Not stated Planned follow-up • 30 days • 6 months • Then every 6 months until registry termination	*N* = 90 treated with cinacalcet, 448 not treated with cinacalcet Inclusion criteria • Treatment for ESRD at a participating center • < 21 years of age prior to enrollment • Receiving dialysis for > 30 days • Complete information on dialysis modality Exclusion criteria • Not stated
Supportive modeling studies		
A Bayesian extrapolation analysis to infer the treatment effect of cinacalcet Data were collected from phase 3 adult studies (20000172, 20000183, and 20000188) along with data from pediatric Studies 20070208 and 20110100.		
Physiologically-based PK modeling Analyses were conducted to predict the PK characteristics of cinacalcet in pediatric subjects < 1 year old and their consistency with those observed in older children (9 months to < 18 years old) and adults.		
PK/PD model of cinacalcet, in adults and pediatrics with sHPT on dialysis Cinacalcet plasma, serum PTH, and serum cCa concentration-time data were obtained from four pediatric clinical studies (20070208, 20110100, 20030227, and 20090005) in CKD subjects with SHPT receiving dialysis, and four adult clinical studies, three of which included ESRD subjects with SHPT and one in healthy subjects treated with single and multiple doses of cinacalcet.		

In the phases 2 and 3 pediatric studies, doses were uptitrated in a stepwise manner every 4 weeks and could have been maintained, reduced, or withheld at all weekly or biweekly visits throughout the dose titration periods, based on plasma PTH and cCa levels, and subject safety information. In addition to those listed, subjects taking grapefruit juice, herbal medications, potent cytochrome P450 3A4 (CYP3A4) inhibitors (e.g., erythromycin, clarithromycin, ketoconazole, itraconazole) or concomitant medications that may prolong the QTc interval (e.g., ondansetron, albuterol) were also excluded from Amgen clinical studies

^a^Before partial clinical hold. ^b^After partial clinical hold. ^c^Czech Republic minimum age is ≥ 2 years of age at enrollment. ^d^Dosing information available from 80/90 (88.9%) subjects

*AE*, adverse events; *cCa*, corrected serum calcium; *CKD*, chronic kidney disease; *ESRD*, end-stage renal disease; *n/a*, not applicable; *NKF-K/DOQI*, National Kidney Foundation Kidney Disease Outcomes Quality Initiative; *PD*, pharmacodynamic; *PK*, pharmacokinetic; *PTH*, parathyroid hormone; *PTx*, parathyroidectomy; *sHPT*, secondary hyperparathyroidism

**Table 2:** Non-Amgen-supported studies of cinacalcet in pediatric subjects

Reference, study type, and objectives	Dosage	Study population	Key findings
Arenas Morales et al [23] Retrospective chart review Primary objective To determine a safe and effective dosing regimen of cinacalcet in the treatment of infants and young children with sHPT that was refractory to standard care Other objectives To examine growth during cinacalcet treatment	Starting dose: 0.4 to 1.1 mg/kg/day (weight-adjusted) Titration: • Titrated on average every 30 days to achieve a decline in the PTH to a goal of 150–300 pg/mL • No set protocol for advancing the cinacalcet dose, although it was generally increased by 50% at each increment Provision: Administered once daily orally or via gastric tube Planned follow-up: 11 courses of cinacalcet treatment	*N* = 10 A retrospective case series of infants and young children with advanced CKD who developed refractory sHPT and were treated with cinacalcet Inclusion criteria • Age < 8 years • PTH > 500 pg/ml for ≥ 30 days • Unresponsive to conventional therapy (high doses of phosphate binders and active vitamin D, calcitriol and/or paricalcitol) Exclusion criteria • Not stated	Efficacy • At the beginning of the observation period, 5 subjects with advanced CKD stage 5 were being managed conservatively off dialysis. Of the remaining, 2 were on peritoneal dialysis and 3 were on chronic hemodialysis. • By the end of the observation period, 3 subjects had been transplanted, 3 were on peritoneal dialysis, 2 remained on hemodialysis, and 1 was with CKD stage 5 off dialysis. • All subjects achieved target goal PTH of 150–300 pg/ml by 8 months and within a median time of 112 days (IQR 56, 259). • Note “rebound” levels of PTH during treatment, especially in those with initial PTH levels > 1000 pg/ml—between 2 and 4 months • 8/10 subjects had improved linear growth during cinacalcet therapy compared to the previous 6 months. Safety • Predominant AEs during cinacalcet therapy were nausea, vomiting, and loss of appetite, especially at high doses of cinacalcet and when taken orally; contributed to poor adherence to cinacalcet in the hemodialysis subjects on their off-dialysis days. • 6/10 subjects experienced Ca < 8.5 mg/dL; none experienced any overt symptom of hypocalcemia such as tremor, paresthesia, or seizure. • Cinacalcet can be effective and safe in controlling PTH as adjuvant therapy with vitamin D sterols and phosphate binders. • One patient died from complications of peritonitis, had been off cinacalcet for over 6 months at the time of death
Alharthi et al. [24] A prospective cohort analysis Primary objective To assess the effect of cinacalcet on intact PTH secretion in children with uncontrolled hyperparathyroidism secondary to CKD CKD-4/5	Starting dose: 0.5 mg/kg/day Titration: Titrated every 2 weeks up to a maximum of 1.5 mg/kg/day Provision: Not stated Planned follow-up: Until the endpoint is achieved (range 3–24 months)	*N* = 28 A prospective, open-label, single-arm interventional study over a period of 24 months. Inclusion criteria • Age ≤ 18 years • Mean baseline PTH ≥ 300 pg/mL • Mean baseline cCa ≥ 8.4 mg/dL • Ca X P product ≥ 65 mg^2^/dL^2^ • Receiving HD or automated peritoneal dialysis (APD) for > 6 months Exclusion criteria • Serum Ca < 8.4 mg/dL • Seizure disorder maintained on anti-convulsant treatment • Hepatic impairment • Hypersensitivity to cinacalcet	Efficacy • Significant reduction in PTH and alkaline phosphatase levels was demonstrated with cinacalcet treatment (mean PTH levels reduced from 1931.76 to 354.25 pg/mL; *P* < 0.001). • Subjects with lower baseline PTH attained target PTH levels quicker than those with initially higher PTH. • No effect on serum Ca, P, or Ca x P product despite an overall significant reduction in PTH levels. • Nine subjects did not achieve the K/DOQI recommended PTH levels at 24 months and are still on treatment. • 2 hemodialysis subjects died of CKD. • Authors recommend cinacalcet use on a wide scale in pediatric CKD-4/5 even at young age. Safety • No cases of symptomatic hypocalcemia or hypophosphatemia were reported.
Platt et al. [25] Retrospective case series Primary objective To assess the effect of cinacalcet on intact PTH secretion in children with uncontrolled hyperparathyroidism secondary to CKD5	Starting dose: 0.4–1.1 mg/kg/day Titration: Doses of cinacalcet were titrated according to serum PTH and AEs such as hypocalcemia. Provision: Not stated Planned follow-up: Not stated	*N* = 6 Case series of continuous cinacalcet use for up to 3 years in subjects with CKD 5. Inclusion criteria • Age ≤ 14 years • PTH > 500 pg/ml for ≥ 30 days • Unresponsive to conventional therapy Exclusion criteria • Not stated	Efficacy • All 6 cases saw a minimum reduction in PTH level of 86% (range 86–98%) over a period of continuous treatment that ranged between 3 months and 2 years. • There was a significant difference in mean PTH level between the 1-month pretreatment level (102.9 pmol/L), and the level during the month in which optimal control was achieved (7.9 pmol/L; *P* = 0.002). • Four subjects required dose increases during treatment; the doses administered ranged from 0.4 to 2.6 mg/kg/day. • One patient showed a decrease in PTH level from 1-month pretreatment to 24 months, but PTH control was lost after 24 months despite increasing doses of cinacalcet. • No significant difference in serum Ca, P, and Ca × P over the duration of treatment Safety • Asymptomatic hypocalcemia was observed in two subjects and hypophosphatemia occurred in three subjects; 1 patient was refractory to treatment, resulting in the discontinuation of cinacalcet for a 5-month period. • Nausea and vomiting not a significant issue
Muscheites et al. [26] A single-center study Primary objective To assess the efficacy and acceptability of cinacalcet for treatment of sHPT pediatric subjects suffering from ESRD presenting with inadequately controlled SHPT	Starting dose 0.25 mg/kg/day (body weight) during a 4-week period. Titration: No titration mentioned, final doses not reported Provision: 30-mg tablets re-pressed into tablets containing 2.5 mg, 5 mg, and 7.5 mg of cinacalcet Planned follow-up: 4 weeks	*N* = 7 A single-center study evaluating cinacalcet administration in pediatric subjects with CKD 5 and sHPT Inclusion criteria • Age ≤ 19 years • PTH > 500 pg/ml • Unresponsive after 2 months conventional therapy (calcitriol and phosphate binders) Exclusion criteria • Not stated	Efficacy • Median serum PTH values decreased rapidly after 4 h and 12 h. • Median PTH decrease amounted to 74% (932 pg/mL at baseline to 199 pg/mL; *P* = 0.028). • Median concentrations of serum Ca showed a significant decrease 4 h after the dose (from 2.69 mmol/L at baseline to 2.38 mmol/L; *P* < 0.05). • Both serum P levels and the Ca × P ion product showed a rapid and sustained decrease, which occurred within the first week of cinacalcet treatment and lasted throughout treatment. Safety • No AEs, e.g., symptomatic hypocalcemia, gastrointestinal symptoms (nausea, vomiting, and diarrhea), were noted. • Two subjects developed asymptomatic hypocalcemia, defined as serum calcium < 2.20 mmol/L, and were treated with calcium or vitamin D.
Silverstein et al. [27] A single-center study Primary objective To assess the efficacy of cinacalcet in pediatric subjects with sHPT (high-turnover bone disease) receiving dialysis	Starting dose: 30 mg/day (once each evening with food) Titration*:* Once every 4 weeks to a maximum of 120 mg once daily for persistent PTH > 500 pg/mL Provision: Not stated Planned follow-up: 3 months	*N* = 9 Retrospective analysis of subjects on chronic dialysis Inclusion criteria • Age 2–21 years • On hemodialysis or peritoneal dialysis for ≥ 6 months • PTH levels ≥ 400 pg/mL for 3 consecutive months Exclusion criteria • Not stated	Efficacy • *n* = 6 remained on 30 mg/day; *n* = 1 increased to 60 mg/day; *n* = 2 increased 120 mg/day. • In subjects on hemodialysis (n = 6), mean (± SD) PTH reduced by 41.7% from 845.73 ± 145.2 pg/mL at 1 month prior to cinacalcet therapy to 493 ± 133.4 pg/mL after 3 months of treatment (*P* = 0.03). • In subjects on peritoneal dialysis (*n* = 3), mean PTH (± SD) reduced by 82.4% from 1518.0 ± 309.5 pg/mL at 1 month prior to therapy to 266.7 ± 93.6 pg/mL after 3 months (*P* = 0.08). • Serum Ca and P levels and Ca x P product were unchanged after 3 months of cinacalcet therapy. Safety • *n* = 3 hemodialysis subjects reported mild nausea that did not require cinacalcet discontinuation. • Other potential adverse symptoms due to cinacalcet not reported.

*AE*, Adverse event; *Ca*, calcium; *CKD*, chronic kidney disease; *h*, hour; *IQR*, interquartile range; *K/DOQI*, National Kidney Foundation Kidney Disease Outcomes Quality Initiative; *P*, phosphate; *PTH*, parathyroid hormone; *SD*, standard deviation; *sHPT*, secondary hyperparathyroidism

## Results

Collectively, 79 subjects received cinacalcet within the Amgen-supported phases 2 and 3 studies, with most subjects (57%) receiving cinacalcet for 16 weeks or longer (demographic details are presented in Table S1a). Across these studies, cinacalcet was initiated at a dose of 0.06–0.29 mg/kg/day (mean 0.16 mg/kg/day). Subjects received a mean cumulative dose of 1794 mg and a maximum weight–adjusted daily dose ranging from 0.1 to 5.7 mg/kg/day. Dosing with cinacalcet in the first phase 3 pediatric study (Study 20070208) was stopped due to a fatality in a cinacalcet-treated individual. The patient was noted to be severely hypocalcemic at the time of death. The cause of death was multifactorial and a contribution of cinacalcet to the death could not be excluded. The program was placed on a partial clinical hold by the US FDA for 14 months following the fatality while changes to cinacalcet dosing were implemented to further minimize the risk of severe hypocalcemia. Study 20070208 was terminated, and changes to cinacalcet dosing were implemented in Study 20130356 and Study 20110100. Dose adjustments of cinacalcet (reduction, withholding, or maintenance) were based on monthly assessments of iPTH and corrected serum calcium levels as well as weekly assessments using ionized calcium. Dose decisions were also based on adverse signs and symptoms (e.g., related to hypocalcemia), investigational product compliance, and administration of medications known to prolong the QT interval.

Adverse events (AEs) were frequent (70/79 patients; 88.6%) and mostly grade 3 or lower (63/79 patients; 79.7%), with the frequency and severity of AEs being similar between the control and test arms of the two controlled studies. No AEs resulted in the discontinuation of cinacalcet. A summary of the treatment-emergent AEs in all pediatric subjects treated with cinacalcet in Amgen-sponsored studies are described in Tables 5 and 6, and those in controlled studies are provided in Table S2. The most common AEs (occurring in > 10% of subjects) were hypocalcemia (18 subjects [22.8%]), vomiting (13 subjects [16.5%]), nausea (12 subjects [15.2%]), systemic hypertension (9 subjects [11.4%]), pyrexia (8 subjects [10.1%]), and muscle spasms (8 subjects [10.1%]). Accounting for cinacalcet exposure and treatment duration, the overall exposure- and follow-up-adjusted subject incidence rate of AEs was 478.5 per 100 subject years and 520.2 per 100 subject years, respectively. Approximately one-third of all subjects reported a serious AE, with the exposure-adjusted incidence of those most commonly reported (by > 1 subject) being device-related infection (10.8 per 100 subject years), peritonitis (7.3 per 100 subject years), overdose, systemic hypertension (each 7.2 per 100 subject years), and complication associated with catheter (7.0 per 100 subject years). Details of safety follow-up and the adjusted incidence of treatment-emergent AEs occurring in ≥ 5% of subjects in any treatment group by system organ class and preferred term are provided in Tables S3 and S4. Most subjects were receiving a phosphate binder and/or a vitamin D sterol at baseline. The proportion of subjects who had a phosphate binder or a vitamin D sterol added to their treatment regimen post-baseline was low and similar between the treatment and control arms. Details on concomitant phosphate binder and vitamin D use are presented in Table S5. The study specific safety data are described below.

**Table 3:** Phases 2 and 3 efficacy data

	20070208		20130356		20110100		20140159
	Cinacalcet (*n* = 22)	PBO (*n* = 21)	Cinacalcet + SOC (*n* = 27)	SOC (*n* = 28)	Cohort 1 (*n* = 7)	Cohort 2 (*n* = 10)	Cinacalcet^a^ (*n* = 13)
PTH (pg/mL)							
Baseline levels Mean (SD) Median (range)	757 (440)	796 (538) 684 (300–2246)	946 (635) 663 (347–2924)	1228 (732) 1123 (300–2701)	1428 (755) 1294 (521–2903)	1207 (598) 1158 (396–2347)	n/a
	676 (309–2407)						
Subjects achieving a ≥ 30% reduction, n/N (%)	12/22 (54.5)	4/21 (19.0)	7/27 (25.9)^b^ 6/27 (22.2)^c^	5/28 (17.9)^b^ 9/28 (32.1)^c^	7/7 (100)	5/10 (50)	4/13 (30.8)^d^ 3/13 (23.1)^e^
Subjects achieving a PTH value ≤300 pg/mL, n/N (%)	6/22 (27.3)	5/21 (23.8)	2/27 (7.4)^f^	5/28 (17. 9)^f^	4/7 (57.1)	5/10 (50)	1/13 (7.7)^e^
Corrected Calcium (mg/dL)							
Baseline levels Mean (SD) Median (range)	9.9 (0.5) 10.1 (8.9–10.8)	9.9 (0.6) 9.8 (9.0–11.3)	9.8 (0.6) 9.7 (8.9–11.8)	9.8 (0.6) 9.8 (8.9–11.0)	10.5 (0.8) 10.3 (9.3–11.5)	9.8 (0.6) 9.8 (8.9–10.9)	9.7 (0.4) 9.6 (9.2–10.4)
LS mean (95% CI) percentage change	− 4.6 (− 8.4, − 0.9)	− 1.0 (− 4.9, 2.9)	− 2.7 (− 5.4, − 0.1)^f^	0.7 (− 1.8, 3.2)^f^	− 0.5 (-3.8, 2.15)^g^ − 3.6 (-10.9, 3.7)^h^	− 3.3 (-0.9,10.5)^g^ 3.6 (-3.3, 11.5)^h^	− 1.4 (− 4.5, 1.5)^i^
Phosphate, mg/dL							
Baseline levels Mean (SD) Median (range)	6.7 (1.8) 6.7 (3.7–12.1)	6.4 (1.5) 6.0 (4.5–10.6)	5.9 (1.4) 5.9 (3.5–10.0)	5.7 (1.1) 5.5 (3.3–8.2)	6.0 (2.2) 5.2 (4.6–10.8)	6.4 (1.3) 6.0 (4.5–9.0)	5.5 (1.1) 5.6 (4.0–7.1)
LS mean (95% CI) percentage change	2.9 (− 8.0, 13.8)	9.3 (− 2.0, 20.6)	13.6 (4.1, 23.0)^f^	− 0.8 (− 10.0, 8.4)^f^	− 10.8 (− 33.9, 10.3)^h^ − 8.7 (− 25.8, 8.5)^j^	− 5.1 (− 31.7, 5.6)^g^ − 9.6 (− 24.7, 13.7)^h^	− 2.1 (− 10.3, 8.2)^i^

^a^Efficacy data from cinacalcet + SOC treated subjects in the parent study (Study 20130356) are presented above therefore, efficacy data here focus only on those subjects (n = 13) who received SOC in the parent study and subsequently received cinacalcet in the extension study. ^b^Weeks 11 and 15 (the US primary endpoint); ^c^Weeks 17 to 20 (US secondary endpoint/global primary endpoint); ^d^During weeks 11 and 15; ^e^ During weeks 23 and 28; ^f^ Weeks 17 to 20 (secondary endpoint); ^g^ Mean (Q1, Q3) presented; ^h^ Mean (Q1, Q3) percent change from baseline at Week 3 presented (n = 17); ^i^ Median (Q1, Q3) presented; ^j^ Mean (Q1, Q3) percentage change from baseline at Week 7 presented (n = 12). Ca, calcium; CI, confidence interval; n/a, not applicable; P, phosphate; PTH, parathyroid hormone; SD, standard deviation; SOC, standard of care

**Table 4:** Titration schema and dosing decisions used in Amgen-supported clinical trials 20070208 and 20130356

Dry weight (kg)	Starting dose (mg)^a,b^	Possible dose titration steps					
		1	2	3	4	5	6
12.5 to 14	2.5	5	10	15	30	30	30
> 14 to 21	2.5	5	10	15	30	60	60
> 21 to 25	2.5	5	10	15	30	60	90
> 25 to 28	5	10	15	30	60	90	90
> 28 to 49	5	10	15	30	60	90	120
> 49 to <75	10	15	30	60	90	120	180
≥ 75	15	30	60	90	120	180	180
Criteria for dosing decisions		Study 20070208^c^			Study 20130356		
Dose maintenance		• PTH: ≥ 150 to < 300 pg/mL • Subject did not meet any criteria for dose reduction or withholding of dose.			• PTH ≥ 150 to < 300 pg/mL • Subject did not meet any criteria for dose reduction or withholding of dose.		
Dose reduction		• PTH ≥ 100 to < 150 pg/mL • cCa ≥ 8.0 to < 8.4 mg/dL • Subject had AE that required dose reduction. • Subject did not meet any criteria for withholding of dose.			• PTH ≥ 100 to < 150 pg/mL • cCa ≥ 8.0 to < 8.4 mg/dL • iCa ≥ 1.00 to < 1.05 mmol/L • Subject did not meet any criteria for withholding of dose.		
Dose withholding		• PTH < 100 pg/mL • cCa < 8.0 mg/dL • Symptoms of hypocalcemia. • Subject had AE that required withholding of dose.			• PTH < 100 pg/mL • cCa < 8.0 mg/dL (via either a central or local assessment^d^) • iCa < 1.00 mmol/L • Symptoms of hypocalcemia • Subject had AE that required withholding of dose. • Temporary administration of concomitant medications (CYP3A4 inhibitors or CYP2D6 substrates) that are known to prolong the QTc interval.		

Dose increases were allowed every 4 weeks. The dose was increased if the plasma PTH was ≥ 300 pg/mL, provided the subject did not meet any criteria for dose maintenance, dose reduction, or dose withholding, and the subject had not reached the maximum allowed dose. In Study 20130356, the dose of cinacalcet was also not increased in subjects who were determined to be noncompliant according to protocol-specified definitions

^a^In Study 20070208, the starting dose was ≤ 0.2 mg/kg/day. ^b^In Study 20130356, the starting dose was 0.2 mg/kg/day rounded down to the nearest protocol-specified dose. ^c^Following the partial hold (prior to study termination), Study 20070208 protocol was amended utilizing the dry weight-based dosing schema for all dose adjustments during the double-blind phase, and the week 30 dry weight for all dose adjustments during the open-label phase. ^d^Total calcium concentration

*AE*, adverse event; *cCa*, corrected serum calcium; *CYP*, cytochrome; *iCa*, ionized calcium; *QTc interval*, corrected QT interval; *PTH*, parathyroid hormone

**Table 5:** Summary of treatment-emergent adverse events in pediatric subjects treated with cinacalcet in Amgen-supported clinical trials

	Study 20070208 (*N* = 28)	Study 20110100 (*N* = 17)	Study 20130356^a^ (*N* = 25)	Study 20140159^b^ (*N* = 9)	Overall (*N* = 79)
All treatment-emergent adverse events, n(%)	25 (89.3)	16 (94.1)	22 (88.0)	7 (77.8)	70 (88.6)
Grade ≥ 2	18 (64.3)	11 (64.7)	14 (56.0)	1 (11.1)	44 (55.7)
Grade ≥ 3	10 (35.7)	8 (47.1)	7 (28.0)	1 (11.1)	26 (32.9)
Grade ≥ 4	1 (3.6)	3 (17.6)	3 (12.0)	0 (0.0)	7 (8.9)
Serious adverse events	12 (42.9)	9 (52.9)	6 (24.0)	1 (11.1)	28 (35.4)
Leading to discontinuation of cinacalcet	0 (0.0)	0 (0.0)	0 (0.0)	0 (0.0)	0 (0.0)
Fatal adverse events	1 (3.6)	0 (0.0)	0 (0.0)	0 (0.0)	1 (1.3)
Treatment-related treatment-emergent adverse events, n(%)	11 (39.3)	3 (17.6)	11 (44.0)	3 (33.3)	28 (35.4)
Grade ≥ 2	6 (21.4)	2 (11.8)	5 (20.0)	0 (0.0)	13 (16.5)
Grade ≥ 3	1 (3.6)	1 (5.9)	1 (4.0)	0 (0.0)	3 (3.8)
Grade ≥ 4	1 (3.6)	0 (0.0)	0 (0.0)	0 (0.0)	1 (1.3)
Serious adverse events	3 (10.7)	0 (0.0)	0 (0.0)	0 (0.0)	3 (3.8)
Leading to discontinuation of cinacalcet	0 (0.0)	0 (0.0)	0 (0.0)	0 (0.0)	0 (0.0)
Fatal adverse events	1 (3.6)	0 (0.0)	0 (0.0)	0 (0.0)^c^	1 (1.3)

^a^Subjects who received cinacalcet in Study 20130356 are counted in the Study 20130356 column; subjects from this cohort who continued to extension Study 20140159 are also counted in the Study 20130356 column. ^b^Subjects who received standard of care in Study 20130356 and received cinacalcet in Study 20140159 are counted in the Study 20140159 column. ^c^Note pooled summary included data from Study 20140159 interim analysis prior to the occurrence of a fatal event deemed not to be related to cinacalcet by the investigator (as reported in the final analyses detailed in the results section)

**Table 6:** Summary of the most commonly reported adverse events within the Amgen-supported Phase 2 and 3 studies

AEs occurring > 30% of subjects, *n* (%)^a^	*N* = 79
Infection and infestations	38 (48.1)
Gastrointestinal disorders	32 (40.5)
Metabolism and nutritional disorders	30 (38.0)
AEs occurring in ≥ 10% of subjects, *n* (%)	
Hypocalcemia	18 (22.8)
Vomiting	13 (16.5)
Nausea	12 (15.2)
Hypertension	9 (11.4)
Pyrexia	8 (10.1)
Muscle spasms	8 (10.1)
Serious AEs reported for > 2 subjects, *n* (%)	
Hypertension	5 (6.3)
Device-related infection	3 (3.8)
Peritonitis	3 (3.8)

^a^Described by organ system class. *AE*, adverse event

Efficacy data from the Amgen-sponsored phases 2 and 3 studies are summarized in Table 3. Overall, the efficacy of cinacalcet in pediatric subjects enrolled in studies before the partial clinical hold was similar across pediatric age groups to that seen in adults. Lower-than-expected efficacy was observed in patients enrolled after the partial clinical hold; dose titration to achieve adequate cinacalcet exposure expected to result in PTH reduction was limited primarily because of frequent dose interruptions and reductions due to more conservative calcium thresholds. Despite limited cinacalcet exposure, twenty-one subjects (75%) achieved a ≥ 30% reduction in PTH after the first dose of cinacalcet during the combined Studies 20130356, 20110100, and 20140159. Furthermore, reductions in PTH were observed in individual subjects with increasing exposure to cinacalcet. Where assessed, no clinically significant results were observed regarding the impact of cinacalcet on bone turnover markers (e.g., serum-specific bone alkaline phosphatase [BALP], type 1 collagen cross-linked N-telopeptide [NTx], amino-terminal propeptide of type 1 collagen [P1NP], and type 1 collagen cross-linked C-telopeptide [CTx]).

### Phases 2 and 3 safety findings

#### Study 20070208

A randomized, double-blind, placebo-controlled Phase 3 study to assess the safety and efficacy of cinacalcet in 43 pediatric subjects with CKD and sHPT receiving dialysis [28].

The mean (standard deviation [SD]) duration of exposure during the double-blind phase was 109.7 (65.9) days in the cinacalcet group and 123.4 (80.4) days in the placebo group. The mean (SD) actual weight-adjusted daily dose of cinacalcet taken during the efficacy assessment phase (EAP; period between weeks 25 and 30, inclusive) was 1.54 (2.04) mg/kg/day. The mean (SD) maximum actual weight-adjusted daily dose of cinacalcet during the double-blind phase was 0.99 (1.26) mg/kg/day. In the double-blind phase, 81.8% of subjects in the cinacalcet group and 85.7% of subjects in the placebo group had at least one AE. The most common AEs in the cinacalcet group were vomiting (31.8% cinacalcet, 23.8% placebo), hypocalcemia (22.7% cinacalcet, 19.0% placebo), and nausea (18.2% cinacalcet, 14.3% placebo). No subjects in the cinacalcet group experienced an AE that led to cinacalcet withdrawal. Nine subjects in both the cinacalcet group (40.9%) and the placebo group (42.9%) had a serious AE. No serious AE was experienced by more than 1 subject. One subject in the cinacalcet group experienced a fatal AE (reported preferred term: cardiopulmonary failure) in the setting of severe hypocalcemia. Although the fatality was determined to be multifactorial, a causal role for hypocalcemia associated with cinacalcet treatment could not be excluded. Due to this fatality, the clinical program was placed on a partial clinical hold for 14 months. Based on a review of the case and in consultation with the Data Monitoring Committee and the FDA, changes were made to the cinacalcet pediatric program to include additional safety measures focused on further minimizing the risk of hypocalcemia in Study 20110100 (restarted after the partial clinical hold) and in new Studies 20130356 and 20140159. Study 20070208 was terminated following the partial clinical hold.

#### Study 20130356

A randomized, multicenter, open-label, controlled (cinacalcet + SOC vs. SOC) Phase 3 study in 55 pediatric subjects with CKD and sHPT receiving dialysis.

The mean (SD) duration of exposure in the cinacalcet + SOC group was 112.8 (41.0) days. The mean (SD) actual weight-adjusted daily dose of cinacalcet taken was 0.3 (0.3) mg/kg/day during weeks 11 to 15, and 0.4 (0.5) mg/kg/day during weeks 17 to 20. The mean (SD) maximum actual weight-adjusted daily dose was 0.6 (0.5) mg/kg/day. During the entire treatment period, 23 of 25 subjects (92.0%) had at least 1 cinacalcet dose withheld or reduced.

Twenty-one subjects (84.0%) in the cinacalcet + SOC group and 17 subjects (56.7%) in the SOC group experienced AEs. The most common AEs were hypocalcemia (6 subjects [24.0%] cinacalcet + SOC, 2 subjects [6.7%] SOC), muscle spasms (3 [12.0%], 2 [6.7%]), nausea (3 [12.0%], 1 [3.3%]), headache (1 [4.0%], 4 [13.3%]), and vomiting (0, 3 [10.0%]). All other AEs were reported for ≤2 subjects in either treatment group. Most subjects had AEs that were grade > 2 in severity, and most were considered not related to study treatment. None of the subjects died or were withdrawn from the study due to an AE. Serious AEs were reported for 4 subjects (16.0%) in the cinacalcet + SOC group and 2 subjects (6.7%) in the SOC group. Each serious AE was reported by a single subject, and none were considered related to study treatment. Review of laboratory parameters, including corrected serum calcium, ionized calcium, and phosphate, revealed no unexpected safety concerns in either treatment group. More subjects in the cinacalcet + SOC group (6 subjects [24.0%]) experienced low corrected serum calcium < 8.4 mg/dL compared with the SOC group (2 subjects [6.9%]); however, the incidences of low corrected serum calcium < 8.0 mg/dL and < 7.5 mg/dL were comparable. The incidence of low ionized calcium was similar between treatment groups.

#### Study 20110100

An open-label, single-arm phase 2 study of cinacalcet in addition to SOC in 18 pediatric subjects aged 28 days to < 6 years.

Seventeen subjects received ≥ 1 cinacalcet dose. Data were assessed before partial clinical hold (cohort 1; *n* = 7) and after partial clinical hold (cohort 2; *n* = 10). The mean total cinacalcet dose administered was 516.9 mg for cohort 1 and 371.3 mg for cohort 2. The higher exposure in cohort 1 was achieved despite a shorter mean (SD) exposure to cinacalcet for cohort 1 (66.0 [50.9] days) compared with cohort 2 (101.2 [42.4] days). The mean (SD) maximum weight–adjusted daily dose was 0.98 (0.94) mg/kg/day and 0.55 (0.31) mg/kg/day for cohorts 1 and 2, respectively. Serious AEs were reported in 9 subjects (52.9%), 4 in cohort 1 and 5 in cohort 2. Complication associated with catheter and systemic hypertension each occurred in 2 subjects (11.8%; 1 subject each per cohort). None of the serious AEs were treatment-related. One subject in cohort 2 experienced a grade 2 serious AE of a seizure 14 days after discontinuation of cinacalcet treatment. The seizure occurred at the end of a hemodialysis session. This event was considered not to be related to cinacalcet treatment by the investigator.

#### Study 20140159

An open-label, phase 3 extension study of cinacalcet in the treatment of sHPT in pediatric subjects with CKD receiving dialysis (extension of Studies 20130356 and 20110100). The final analyses included 27 subjects rolled over from the parent Study 20130356 and 1 subject rolled over from Study 20110100. Of the 27 subjects from Study 20130356, 14 subjects previously received cinacalcet and SOC in the parent study (hereinafter referred to as the Study 20130356 cinacalcet + SOC group), and 13 subjects previously received SOC alone in the parent study (referred to as Study 20130356 SOC hereafter).

The mean (SD) maximum weight-adjusted exposure to cinacalcet during the extension study was 0.78 (0.66) mg/kg/day, across 169.8 (52.4) days. Nine subjects (32.1%; 6 subjects from Study 20130356 cinacalcet + SOC, 2 subjects from Study 20130356 SOC, and 1 subject from Study 20110100 cinacalcet + SOC) had at least 1 serious adverse event and at least 1 grade ≥3 adverse event. Nine subjects (32.1%; 4 subjects from Study 20130356 cinacalcet + SOC and 5 subjects from Study 20130356 SOC) had adverse events that were considered treatment related by the investigator. A 2-year 7-month-old boy died; it was determined by autopsy that the death was due to bronchopneumonia and acute exacerbation of chronic pyelonephritis. According to the investigator, there was no reasonable possibility that the fatal event was related to cinacalcet.

### Phase 1 pharmacokinetic studies

Two phase 1 pharmacokinetic (PK) studies (Study 20090005 and Study 20030227) have been conducted and the PK findings published. Study 20090005 was an open-label, single-dose study (0.25 mg/kg) of cinacalcet in 14 pediatric subjects aged 28 days to < 6 years with CKD receiving dialysis [29]. Study 20030227 was an open-label study of a single dose (15 mg) of cinacalcet in 12 pediatric subjects age 6 to < 18 years with CKD receiving dialysis [30].

### Safety and efficacy data from observational studies

#### Study 20090198

A retrospective chart review to evaluate biochemical markers and safety in children < 6 years of age with sHPT and CKD receiving dialysis at the time of initiation of cinacalcet treatment.

#### Safety

This study included a total of 23 subjects on dialysis who received cinacalcet. Nineteen of the 23 subjects (83%) received cinacalcet as a crushed tablet. The route of administration was oral for 15 subjects (65%), by nasogastric tube for 7 subjects (30%), and through a percutaneous endoscopic gastrostomy for 1 subject (4%). The mean (SD) duration of exposure was 274.17 (245.53) days, with an exposure range of 34–1036 days. The mean (SD) initial weight-adjusted daily dose was 0.81 (0.54) mg/kg/day (median [range] 0.61 [0.1, 1.9] mg/kg/day). The mean (SD) maximum weight–adjusted daily dose over the course of treatment was 1.87 (1.30) mg/kg/day (median [range] 1.40 [0.4, 5.6] mg/kg/day). Seventeen subjects (74%) had at least 3 months of cinacalcet treatment. A review of dose administration by individual subject showed variable dosing regimens with doses ranging from 2.5 to 60 mg. The frequency of administration changed over time for some subjects, although the most common frequency was once daily. Two subjects had adverse drug reactions (i.e., cinacalcet-related AEs): decreased level of consciousness and intermittent mild hypocalcemia; the decreased level of consciousness event occurred in the setting of marked hypocalcemia and was considered serious.

### Efficacy

#### PTH

The proportion of subjects with ≥ 30% reduction in PTH from baseline was 33% (6 of 18 subjects), 42% (5 of 12 subjects), and 58% (7 of 12 subjects) of subjects at months 1, 2, and 3, respectively. Mean PTH concentrations decreased from baseline at every time point, except month 4. The mean (SD) decreases at months 1, 2, and 3 were − 86.8 (815.9), − 533.3 (1055.3), and − 473.5 (871.7) pg/mL, respectively.

#### Calcium

In general, mean serum calcium concentrations remained within normal limits during treatment and ranged from 8.72 (1.11) mg/dL at month 1 to 9.76 (1.13) mg/dL at month 2. Mean decreases from baseline in total calcium were observed at every time point, except month 2. The mean (SD) changes from baseline at months 1, 2, and 3 were − 0.6 (0.8), 0.4 (1.3), and − 0.2 (0.8) mg/dL, respectively. The maximum mean (SD) decreases were at months 1 and 5 (− 0.6 [0.8] mg/dL; − 0.6 [0.7]mg/dL), respectively.

#### Phosphate

Mean decreases from baseline in phosphate were observed at every time point. The largest mean (SD) decrease from baseline was − 1.48 (2.32) mg/dL observed at month 2. The mean (SD) percent decrease from baseline at this same time point was − 20.79% (30.52%). Unexpectedly, the overall group mean percent change from baseline increased at months 1, 5, 6, and 7. This increase is likely skewed by substantial percent increases over baseline for 2 subjects. Median percent decreases in phosphorus from baseline were observed at all time points except month 1 and ranged in value from − 5.60% at month 4 to − 24.24% at month 2. In general, mean serum phosphate concentrations remained within normal limits (i.e., 4.5 to 8.0 mg/dL) during treatment.

#### Study 20120116

A prospective cohort study to describe the use and safety of cinacalcet in pediatric subjects receiving dialysis in the North American Pediatric Renal Trials and Collaborative Studies (NAPRTCS) registry.

This study evaluated 538 subjects on dialysis, including 90 who received cinacalcet and 448 who did not receive cinacalcet. Data collected from this study contributed to the evaluation of the safety rather than the efficacy of cinacalcet. The incidence of AEs of interest (hypocalcemia, seizure, and infection [requiring hospitalization]) was estimated separately in cinacalcet-treated and untreated subjects enrolled in the pediatric dialysis registry. Patients were followed for up to 3 years. Patients were censored at 3 years, transplant, death, loss to follow-up, or if no longer participating in NAPRTCS, whichever occurred first.

#### Hypocalcemia

The incidence of subjects who were treated for or had a modification of treatment for hypocalcemia was similar between subjects who received cinacalcet and subjects who did not. Ten of 90 subjects (11.1%) who received cinacalcet were treated for or had a modification of treatment for hypocalcemia a total of 28 times during the study; 20 therapy modification episodes involved a change in cinacalcet therapy. In subjects not receiving cinacalcet, 48 of 448 (10.7%) had a total of 95 hypocalcemia treatments or modification of treatment for hypocalcemia.

#### Seizure

The incidence of seizure was similar between subjects receiving cinacalcet and subjects not receiving cinacalcet. In subjects receiving cinacalcet, 5 of 90 subjects (5.6%) reported 5 seizure episodes; 4 subjects experienced seizures while taking cinacalcet, 1 subject experienced a seizure while not actively taking cinacalcet. The incidence rate (95% CI) of seizures per follow-up year was 0.04 (0.01, 0.08) in participants receiving cinacalcet and 0.08 (0.06, 0.11) in participants not receiving cinacalcet.

#### Infection

Bacterial infections were reported in 89% (34 of 38) of the hospitalizations in the cinacalcet group and in 65% (116 of 179) of the hospitalizations in the group that did not receive cinacalcet. Viral infections were reported in 8% (3 of 38) of the hospitalizations in the cinacalcet group and 22% (39 of 179) of the hospitalizations in the group that did not receive cinacalcet. Three of the 8 subjects who died in this study had received cinacalcet. Of these three subjects, one subject with focal segmental glomerulosclerosis collapsed and was unable to be resuscitated. The cause of death was noted as “unknown”. Cinacalcet dosing information was not available, and the subject was not taking cinacalcet at last follow-up. The second subject died of hemorrhagic pancreatitis. The subject received therapy with cinacalcet for 5 months and had discontinued cinacalcet therapy 7 months before death. The third subject had acute lymphoblastic leukemia-multiple complications; the subject was receiving cinacalcet at last follow-up (86 days before death).

### Bayesian extrapolation analysis

Innovative study designs and advanced analysis methods including Bayesian approaches are encouraged by the FDA and EMA to improve efficiency and optimize pediatric drug development by minimizing exposure of children to studies and by addressing feasibility issues such as the limited available pediatric population. Accordingly, a Bayesian hierarchical model was used to synthesize information from the adult cinacalcet Studies 20000172, 20000183, and 20000188 and the pediatric cinacalcet Studies 20070208 and 20110100 (specifically, subjects enrolled before the partial clinical hold) to make statistical inference on the treatment effect of cinacalcet in the pediatric population. Bayesian extrapolation demonstrated a higher proportion of pediatric subjects in the cinacalcet group who achieved a ≥ 30% reduction from baseline in mean PTH compared with the placebo-controlled group in both the overall pediatric population and in the younger age group between 28 days and < 6 years of age.

### Physiologically-based PK modeling

Physiologically-based PK modeling analyses were conducted to predict the PK characteristics of cinacalcet in pediatric subjects < 1 year old and their consistency with those observed in older children (1 year to < 18 years old) and adults. Physiologically-based PK simulations projected that the average, weight-normalized oral clearance of cinacalcet in subjects 28 days to 1 year and in subjects 28 days to < 18 years varied < 1.5-fold. At the lowest dose analyzed (0.2 mg/kg), the average *C*_max_, AUC, and weight-normalized oral clearance of cinacalcet were projected to vary < 1.5-fold in subjects 28 days to 1 year, supporting its selection as the weight-based starting dose for subjects aged 28 days to 1 year. Thus, cinacalcet PK data are similar between pediatric and adult subjects with CKD and secondary HPT receiving dialysis and between pediatric age groups (28 days to < 6 years and 6 years to < 18 years).

### PK/PD model of cinacalcet, in adults and pediatric patients with sHPT on dialysis [31]

Cinacalcet PK parameters were described by a two-compartment linear model with delayed first-order absorption-elimination (apparent clearance = 287.7 L/h). Simulations suggested that pediatric starting doses (1, 2.5, 5, 10, and 15 mg) would provide PK exposures less than or similar to a 30-mg adult dose. The titrated dose simulations suggested that the mean (prediction interval) proportion of pediatric and adult subjects achieving ≥ 30% reduction in PTH from baseline at week 24 was 49% (36%, 62%) and 70% (63%, 77%), respectively. Additionally, the mean (confidence interval) proportion of pediatric and adult subjects achieving corrected calcium ≤ 8.4 mg/dL at week 24 was 8% (2%, 18%) and 24% (18%, 31%), respectively. Model-based simulations showed that the pediatric cinacalcet starting dose (0.2 mg/kg/day, based on the subject’s dry weight, rounded down to the lowest protocol-specified dose [PSD]), titrated to effect, would provide the desired pharmacodynamic efficacy (PTH suppression > 30%) while minimizing safety concerns (hypocalcemia).

### Non-Amgen-supported studies

Five non-Amgen-supported clinical studies of cinacalcet use in pediatric subjects with CKD and sHPT receiving dialysis were identified and are described in Table 2. These studies comprised two single-center studies, one retrospective chart review, one retrospective case series, and one prospective cohort analysis. Overall, these studies reported safety and efficacy data from 60 children (age 0.5–19 years; see Table S1b) following cinacalcet treatment with starting dose ranging from 0.25 to 1.1 mg/kg/day. No cases of overt symptoms of hypocalcemia such as tremor, paresthesia, or seizure were reported. Across these studies, cinacalcet reduced PTH levels by 60 to 98% from baseline values. Interestingly, all children with refractory sHPT attained K/DOQI PTH target goal (150–300 pg/ml) within 8 months of cinacalcet initiation [23]. Patients with lower baseline PTH were reported to attain target PTH levels quicker than those with high PTH levels. Furthermore, a “rebound” in PTH levels was reported in patients with a baseline PTH > 1000 pg/mL. One study demonstrated improved linear growth in 80% (8/10) of patients during 6 months of cinacalcet treatment [23]. Overall, findings on changes in serum calcium and phosphate levels were inconclusive.

## Discussion

Treatment strategies for sHPT in children need to account for their higher calcium requirements and bone development. KDIGO recognizes that children may be uniquely vulnerable to calcium restriction and provides guidelines to account for the higher calcium requirements of the growing skeleton, recommending serum calcium be maintained within the age-appropriated normal range and that phosphate-lowering treatment selection be informed by the serum calcium level. Childhood and adolescence are critical periods for bone development with the approximate calcium content of the skeleton increasing from 25 g at birth to 1000 g in adults, and approximately 25% of total skeletal mass is laid down during the 2-year interval of peak height velocity at adolescence [32]. Furthermore, medication recommendations must consider the fact that the capacity to metabolize drugs in children varies throughout development of CYP enzymes and is completed by approximately 6 years of age [30]. Given the extensive weight range common in children and our conservative dosing requirements, a weight-adjusted dosing schema was deemed warranted for cinacalcet.

Six interventional studies have been conducted to obtain information about the safety and efficacy of cinacalcet in pediatric subjects. We have assembled the data here to provide pediatric nephrologists with information that can improve patient care.

The clinical data that have been generated are generally consistent with model-based simulations that showed the pediatric cinacalcet starting dose (0.2 mg/kg/day [based on subject’s dry weight at enrolment, rounded down to the lowest PSD]), when titrated to effect, would provide the desired pharmacodynamic efficacy (PTH suppression > 30%) while minimizing safety concerns (hypocalcemia) [31]. All phases 2 and 3 clinical studies reported here used weight-based dosing to minimize exposure variability between subjects at different developmental stages. Adjusting cinacalcet doses to correct and maintain PTH within target levels [15] and to maintain calcium concentrations within age-appropriate levels [13, 14] was an effective treatment strategy that also showed an AE profile consistent with the known safety profile of cinacalcet in the treatment of adults with sHPT as listed in the prescribing information [20].

The effect of cinacalcet on PTH varied across studies. Overall, 7.4 to 57.1% of subjects who received cinacalcet in an Amgen Inc. clinical trial attained PTH levels within the K/DOQI target range and 22.2 to 70.6% observed a ≥ 30% reduction in PTH. In addition, significant reductions in PTH were demonstrated in all non-Amgen-supported studies. Furthermore, the real-world effectiveness and safety data from the retrospective chart review and the prospective registry supported the safety and efficacy findings from the clinical studies. This real-world data demonstrated that cinacalcet treatment had expected effects on biochemical markers of sHPT and no unexpected safety concerns observed, despite varied doses (mean range 0.8 to 1.9 mg/kg/day) and frequency of administration among children < 6 years of age with CKD and sHPT. The variability in PTH response may, in part, be due to the success of the dose titration. As mentioned above, in Study 20130356, the dose titration rules were conservative, and dose titration was limited based on calcium levels. This may have contributed to a smaller proportion of subjects achieving target PTH levels or at least a 30% reduction in PTH in this study. Interestingly, no significant trends in changes in calcium or phosphate levels were observed in either the Amgen or non-Amgen-supported studies. Serum calcium levels need to be closely monitored and managed during treatment with cinacalcet in children to minimize the risk of hypocalcemia.

Although not evaluated here, bone-related complications and vascular or other soft tissue calcifications are clinically important aspects of CKD-MBD. High turnover skeletal lesions in sHPT cause disproportionate bone loss that leads to thinning of cortical tissue, reductions in cortical bone mass, and an increased risk of skeletal fracture both in adults and in children with advanced CKD [4, 11, 33–37]. Similarly, alterations in the epiphyseal growth plate cartilage architecture can adversely affect endochondral bone formation and impair linear bone growth [38] contributing to growth retardation and to skeletal deformity in children with CKD. Data from one non-Amgen-sponsored study has suggested that cinacalcet may improve linear growth [23]; however, further validation of this finding is required. The addition of cinacalcet to SOC may offer protection against these bone-related complications. The recent BONAFIDE study showed that high rates of bone formation and several biochemical markers of high-turnover bone disease decreased toward normal as PTH was reduced during the treatment of sHPT with cinacalcet [39], potentially due to CaSR activation in osteoblasts promoting bone turnover [40]. However, these benefits should be viewed cautiously as concurrent treatment with vitamin D sterols was allowed throughout the BONAFIDE study, and their impact on bone turnover was not determined [39]. Despite advances in CKD-MBD management, subjects with CKD-MBD receiving maintenance hemodialysis, including children, still experience cardiovascular (CV) morbidity and mortality. High serum phosphorus contributes to the development of vascular calcifications in subjects undergoing dialysis [41, 42]. Indeed, due to this high CV morbidity in children with ESRD, the American Heart Association recently recommended that mineral metabolism abnormalities (i.e., high phosphorus level, sHPT) should be screened for and treated to prevent coronary artery calcification, and children should undergo regular echocardiographic monitoring for LVH [43]. Whether cinacalcet provides CV benefit remains to be fully elucidated. One systematic review of calcimimetics for sHPT in CKD patients concluded that cinacalcet had uncertain effects on CV mortality for CKD stage 5 adult patients on dialysis [44]. Additionally, the EVOLVE study failed to show significant survival benefit for adult patients with cinacalcet in the unadjusted primary analysis [45]. However, secondary analyses of EVOLVE data demonstrated lower rates of CV death and major CV events associated with treatment-induced reductions in serum FGF23 [46]. Furthermore, in two large cohort studies, cinacalcet was shown to reduce overall and CV mortality in adult patients with PTH > 300 pg/mL [47] and adult patients with moderate sHPT (PTH 300–599 pg/mL), younger age, and without diabetes [48].

This review of cinacalcet use in pediatrics has several strengths and limitations. Children with sHPT represent a very small population of subjects that frequently receive kidney transplants, creating difficulty for clinical trials in pediatric dialysis to enroll large numbers of patients, and to evaluate hard outcomes that are present in adult studies such as CV morbidity or death. Furthermore, trial data are limited by subjects discontinuing treatment for transplant. In turn, to obtain data in this patient population, it was important to consider information from multiple sources and conduct novel analyses to evaluate dosing, safety, and efficacy. We have provided the largest pediatric clinical trial data collection assessing the safety and efficacy of cinacalcet use in children with sHPT receiving dialysis in one report. Furthermore, these data are supported with findings from a comprehensive review of clinical trial and real-world data on cinacalcet use in pediatric subjects. Whereas the provision of all available data to pediatric nephrologists is warranted, the conclusions are limited by the fact that data are assessed from multiple sources and are not directly comparable. Additionally, the theoretical corrected calcium inclusion criteria used in the randomized trials (corrected calcium ≥ 8.8 mg/dL) may not reflect real-world treatment decision values guided by the summary of product characteristics and KDIGO and K/DOQI guidelines which suggest the corrected calcium should be in the upper range of, or above, the age-specified reference range prior to administration of the first dose of cinacalcet [13, 15, 22]. Furthermore, whether vitamin D sterols utilized with calcium-free phosphate binders diminish episodes of hypercalcemia was not assessed and warrants further clarification. However, the frequency of hypocalcemia in subjects receiving cinacalcet was similar between Studies 20070208 (22.7%, Table S4) and 20130356 (24.0%, Table S4) despite the presence of 2-fold more subjects in 20070208 (68.2%, Table S5) using calcium-containing phosphate binders than in 20130356 (33.3%, Table S5). Finally, due to study designs, no conclusions can be drawn on the potential impact of cinacalcet on bone density, fracture risk, and CV comorbidities.

In summary, this manuscript contains the most comprehensive review of cinacalcet usage in pediatric patients to date. A patient registry in Europe will provide additional information about the occurrence of hypocalcemia and its management in children receiving cinacalcet. In the meantime, data presented here together with recently published European guidelines [49] will aid in the management of pediatric patients with sHPT on dialysis.

## Electronic supplementary material

ESM 1 (DOCX 55.5 kb)

ESM 2 (PPTX 63 kb)
