# Supplementary material for: Cinacalcet studies in pediatric subjects with secondary hyperparathyroidism receiving dialysis
Source: Pediatr Nephrol. 2020 May 4;35(9):1679–97. doi: 10.1007/s00467-020-04516-4 (PMC7385021; doi:10.1007/s00467-020-04516-4)
Supplement: Supplementary file 1 — (DOCX 55.5 kb) [file 467_2020_4516_MOESM1_ESM.docx]

# **Supplementary Material**

## **Table S1a.** Demographics of pediatric subjects receiving cinacalcet in Amgen-supported clinical trials

|  | **Study**  **20070208**  **(N = 28)** | **Study**  **20110100**  **(N = 17)** | **Study**  **20130356^a^**  **(N = 25)** | **Study**  **20140159^b^**  **(N = 9)** | **Overall**  **(N = 79)** |
| --- | --- | --- | --- | --- | --- |
| Age, years  Mean (SD)  Median (range) | 13.2 (3.5)  14.5 (6.0, 18.0) | 2.9 (1.3)  3.0 (1.0, 5.0) | 13.1 (3.8)  15.0 (6.0, 17.0) | 14.3 (3.1)  15.0 (9.0, 18.0) | 11.1 (5.4)  13.0 (1.0, 18.0) |
| Age group, n (%)  28 days – < 6 years  6 years – < 12 years  12 years – < 18 years | 0 (0.0)  8 (28.6)  20 (71.4) | 17 (100.0)  0 (0.0)  0 (0.0) | 0 (0.0)  8 (32.0)  17 (68.0) | 0 (0.0)  2 (22.2)  7 (77.8) | 17 (21.5)  18 (22.8)  44 (55.7) |
| Sex, n (%)  Male  Female | 15 (53.6)  13 (46.4) | 11 (64.7)  6 (35.3) | 14 (56.0)  11 (44.0) | 4 (44.4)  5 (55.6) | 44 (55.7)  35 (44.3) |
| Race, n (%)  Black (or African American)  Multiple  White, Native Hawaiian or other Pacific Islander  White  Other | 7 (25.0)  0 (0.0)  0 (0.0)  20 (71.4)  1 (3.6) | 2 (11.8)  0 (0.0)  0 (0.0)  15 (88.2)  0 (0.0) | 5 (20.0)  1 (4.0)  1 (4.0)  17 (68.0)  2 (8.0) | 1 (11.1)  0 (0.0)  0 (0.0)  8 (88.9)  0 (0.0) | 15 (19.0)  1 (1.3)  1 (1.3)  60 (75.9)  3 (3.8) |

^a^Subjects who received cinacalcet in Study 20130356 are counted in the Study 20130356 column; subjects from this cohort who continued to extension Study 20140159 are also counted in the Study 20130356 column. ^b^Subjects who received standard of care in Study 20130356 and received cinacalcet in Study 20140159 are counted in the Study 20140159 column.

SD, standard deviation

## **Table S1b.** Demographics of pediatric subjects receiving cinacalcet in non-Amgen-supported studies

|  | **Arenas Morales et al**  **(N = 10)** | **Alharthi et al  (N = 28)** | **Platt et al  (N = 6)** | **Muscheites et al**  **(N = 7)** | **Silverstein et al  (N = 9)** |
| --- | --- | --- | --- | --- | --- |
| Age, years  Mean (SD)  Median (range) | 2.3 (2.0)  1.5 (0.5–7.5) | N/A  N/A (0.75–14) | 5.7 (5.8)  2.8 (0.9–14) | N/A  17.0 (1.1–19.0) | 14.5 (N/A)  N/A (7.5–17.5) |
| Age group, n (%)  28 days – < 6 years  6 years – < 12 years  12 years – < 18 years | 9 (90)  1 (10)  0 (0) | N/A  N/A  N/A | 4 (66.7)  0 (0)  2 (33.3) | N/A  N/A  N/A | N/A  N/A  N/A |
| Sex, n (%)  Male  Female | 7 (70)  3 (30) | 10 (35.7)  18 (64.3) | 5 (83.3)  1 (16.7) | 3 (42.9)  4 (57.1) | 6 (66.7)  3 (33.3) |

N/A, not available

## **Table S2.** Comparison of treatment-emergent adverse events in pediatric subjects within the treatment and control arms of Amgen supported cinacalcet randomized-controlled clinical trials

|  | **Study 20070208**  **Double-blind Phase** | | **Study 20130356** | |
| --- | --- | --- | --- | --- |
|  | **Control^a^**  **(N = 21)** | **Cinacalcet**  **(N = 22)** | **Control^a^**  **(N = 30)** | **Cinacalcet**  **(N = 25)** |
| All treatment-emergent adverse events, n (%)  Grade ≥ 2  Grade ≥ 3  Grade ≥ 4  Serious adverse events  Leading to discontinuation of investigational product  Fatal adverse events | 18 (85.7)  16 (76.2)  10 (47.6)  2 (9.5)  9 (42.9)  2 (9.5)  0 (0.0) | 18 (81.8)  13 (59.1)  7 (31.8)  1 (4.5)  9 (40.9)  0 (0.0)  1 (4.5) | 17 (56.7)  10 (33.3)  3 (10.0)  0 (0.0)  2 (6.7)  0 (0.0)  0 (0.0) | 21 (84.0)  14 (56.0)  4 (16.0)  3 (12.0)  4 (16.0)  0 (0.0)  0 (0.0) |
| Treatment-related treatment-emergent adverse events, n (%)  Grade ≥ 2  Grade ≥ 3  Grade ≥ 4  Serious adverse events  Leading to discontinuation of investigational product  Fatal adverse events | 11 (52.4)  4 (19.0)  2 (9.5)  1 (4.8)  2 (9.5)  1 (4.8)  0 (0.0) | 8 (36.4)  3 (13.6)  1 (4.5)  1 (4.5)  2 (9.1)  0 (0.0)  1 (4.5) | 0 (0.0)  0 (0.0)  0 (0.0)  0 (0.0)  0 (0.0)  0 (0.0)  0 (0.0) | 8 (32.0)  4 (16.0)  1 (4.0)  0 (0.0)  0 (0.0)  0 (0.0)  0 (0.0) |

^a^Study 20070208 control = placebo; Study 20130356 control = standard of care

## **Table S3.** Safety follow-up adjusted incidence rates of treatment-emergent adverse events occurring in ≥ 5% of pediatric subjects receiving cinacalcet in Amgen supported clinical trials by system organ class and preferred term

| **System Organ Class**  **Preferred Term** | **Study**  **20070208**  **(N = 28)**  **n (%) / e [r]** | **Study**  **20110100**  **(N = 17)**  **n (%) / e [r]** | **Study**  **20130356**  **(N = 25)**  **n (%) / e [r]** | **Study**  **20140159**  **(N = 9)**  **n (%) / e [r]** | **Overall**  **(N = 79)**  **n (%) / e [r]** |
| --- | --- | --- | --- | --- | --- |
| Number of subjects reporting treatment-emergent adverse events | 25 (89.3) / 5.2 [476.6] | 16 (94.1) / 1.8 [896.3] | 22 (88.0) / 4.0 [545.5] | 7 (77.8) / 2.4 [292.5] | 70 (88.6) / 13.5 [520.2] |
| Gastrointestinal disorders  Abdominal pain  Constipation  Diarrhea  Nausea  Vomiting | 5 (17.9) / 12.1 [41.2]  1 (3.6) / 13.0 [7.7]  2 (7.1) / 13.0 [15.4]  7 (25.0) / 11.2 [62.3]  8 (28.6) / 11.2 [71.4] | 0 (0.0) / 5.4 [0.0]  1 (5.9) / 5.4 [18.6]  3 (17.6) / 4.6 [64.6]  1 (5.9) / 5.2 [19.2]  4 (23.5) / 4.7 [84.8] | 1 (4.0) / 12.1 [8.3]  1 (4.0) / 11.9 [8.4]  2 (8.0) / 11.5 [17.4]  4 (16.0) / 11.3 [35.3]  1 (4.0) / 11.9 [8.4] | 1 (11.1) / 4.0 [24.9]  1 (11.1) / 4.0 [24.8]  0 (0.0) / 4.2 [0.0]  0 (0.0) / 4.2 [0.0]  0 (0.0) / 4.2 [0.0] | 7 (8.9) / 33.7 [20.8]  4 (5.1) / 34.3 [11.7]  7 (8.9) / 33.3 [21.0]  12 (15.2) / 31.9 [37.6]  13 (16.5) / 31.9 [40.7] |
| General disorders and administration site conditions  Pyrexia | 3 (10.7) / 12.5 [24.0] | 3 (17.6) / 4.8 [62.2] | 2 (8.0) / 12.0 [16.7] | 0 (0.0) / 4.2 [0.0] | 8 (10.1) / 33.5 [23.9] |
| Infections and infestations  Device related infection  Influenza  Nasopharyngitis  Peritonitis  Upper respiratory tract infection | 2 (7.1) / 12.9 [15.5]  4 (14.3) / 12.0 [33.3]  3 (10.7) / 12.3 [24.4]  2 (7.1) / 12.3 [16.3]  0 (0.0) / 13.1 [0.0] | 1 (5.9) / 5.0 [20.1]  1 (5.9) / 5.2 [19.1]  0 (0.0) / 5.4 [0.0]  1 (5.9) / 5.3 [18.8]  4 (23.5) / 4.5 [89.7] | 2 (8.0) / 11.5 [17.4]  0 (0.0) / 12.5 [0.0]  2 (8.0) / 11.6 [17.3]  2 (8.0) / 11.3 [17.7]  0 (0.0) / 12.5 [0.0] | 0 (0.0) / 4.2 [0.0]  0 (0.0) / 4.2 [0.0]  1 (11.1) / 3.7 [27.2]  0 (0.0) / 4.2 [0.0]  0 (0.0) / 4.2 [0.0] | 5 (6.3) / 33.5 [14.9]  5 (6.3) / 33.9 [14.8]  6 (7.6) / 33.0 [18.2]  5 (6.3) / 33.0 [15.1]  4 (5.1) / 34.1 [11.7] |
| Metabolism and nutrition disorders  Hypocalcemia | 9 (32.1) / 10.2 [88.5] | 1 (5.9) / 5.4 [18.6] | 7 (28.0) / 10.3 [68.1] | 1 (11.1) / 3.7 [27.2] | 18 (22.8) / 29.5 [61.0] |
| Musculoskeletal and connective tissue disorders  Muscle spasms  Musculoskeletal stiffness | 3 (10.7) / 12.5 [24.0]  2 (7.1) / 12.6 [15.9] | 0 (0.0) / 5.4 [0.0]  0 (0.0) / 5.4 [0.0] | 3 (12.0) / 10.6 [28.2]  1 (4.0) / 12.0 [8.4] | 2 (22.2) / 3.8 [52.1]  1 (11.1) / 4.1 [24.5] | 8 (10.1) / 32.4 [24.7]  4 (5.1) / 34.1 [11.7] |
| Nervous system disorders  Headache  Paresthesia  Tremor | 4 (14.3) / 11.6 [34.5]  3 (10.7) / 12.5 [23.9]  3 (10.7) / 12.0 [24.9] | 0 (0.0) / 5.4 [0.0]  0 (0.0) / 5.4 [0.0]  0 (0.0) / 5.4 [0.0] | 1 (4.0) / 12.1 [8.2]  1 (4.0) / 11.8 [8.5]  0 (0.0) / 12.5 [0.0] | 1 (11.1) / 4.1 [24.5]  1 (11.1) / 4.1 [24.5]  1 (11.1) / 3.7 [26.8] | 6 (7.6) / 33.2 [18.1]  5 (6.3) / 33.8 [14.8]  4 (5.1) / 33.7 [11.9] |
| Vascular disorders  Hypertension  Hypotension | 4 (14.3) / 11.8 [34.0]  3 (10.7) / 12.6 [23.7] | 4 (23.5) / 4.8 [82.9]  1 (5.9) / 5.1 [19.6] | 1 (4.0) / 12.2 [8.2]  0 (0.0) / 12.5 [0.0] | 0 (0.0) / 4.2 [0.0]  0 (0.0) / 4.2 [0.0] | 9 (11.4) / 33.0 [27.3]  4 (5.1) / 34.4 [11.6] |

Coded using MedDRA version v19.0. N, number of subjects in the analysis set; n, number of subjects reporting at least 1 occurrence of an adverse event. Percentages are based on N. e, sum across all subjects, the total time to first event or total safety follow-up if no event (years); r, Safety follow-up adjusted subject rate per 100 subject years (n/e*100).

## **Table S4.** Safety follow-up adjusted incidence rates of treatment-emergent adverse events occurring in ≥ 5% of subjects in any treatment group by system organ class and preferred term

| **System Organ Class**  **Preferred Term** | **Study 20070208**  **Double-blind Phase** | | **Study 20130356** | |
| --- | --- | --- | --- | --- |
|  | **Control^a^**  **(N = 21)**  **n (%) / e [r]** | **Cinacalcet**  **(N = 22)**  **n (%) / e [r]** | **Control^a^**  **(N = 30)**  **n (%) / e [r]** | **Cinacalcet**  **(N = 25)**  **n (%) / e [r]** |
| Number of subjects reporting treatment-emergent adverse events | 18 (85.7) / 2.4 [759.2] | 18 (81.8) / 3.8 [478.1] | 17 (56.7) / 5.8 [293.9] | 21 (84.0) / 3.4 [621.6] |
| Gastrointestinal disorders  Abdominal pain  Abdominal pain upper  Constipation  Diarrhea  Nausea  Vomiting | 3 (14.3) / 8.9 [33.6]  0 (0.0) / 9.7 [0.0]  3 (14.3) / 8.7 [34.3]  4 (19.0) / 8.8 [45.6]  3 (14.3) / 8.8 [34.0]  5 (23.8) / 7.8 [64.3] | 3 (13.6) / 8.2 [36.5]  1 (4.5) / 8.6 [11.7]  1 (4.5) / 8.6 [11.6]  2 (9.1) / 8.6 [23.3]  4 (18.2) / 8.2 [48.9]  7 (31.8) / 7.5 [93.4] | 1 (3.3) / 10.4 [9.6]  2 (6.7) / 10.6 [18.9]  0 (0.0) / 10.9 [0.0]  2 (6.7) / 10.3 [19.3]  1 (3.3) / 10.7 [9.4]  3 (10.0) / 9.7 [30.8] | 1 (4.0) / 8.3 [12.0]  1 (4.0) / 8.4 [11.9]  0 (0.0) / 8.7 [0.0]  1 (4.0) / 8.3 [12.0]  3 (12.0) / 8.1 [36.8]  0 (0.0) / 8.7 [0.0] |
| General disorders and administration site conditions  Chills  Pain  Pyrexia | 2 (9.5) / 9.1 [22.1]  2 (9.5) / 9.1 [21.9]  4 (19.0) / 8.2 [48.6] | 1 (4.5) / 8.3 [12.1]  0 (0.0) / 8.7 [0.0]  1 (4.5) / 8.6 [11.7] | 0 (0.0) / 10.9 [0.0]  0 (0.0) / 10.9 [0.0]  1 (3.3) / 10.8 [9.3] | 1 (4.0) / 8.6 [11.6]  0 (0.0) / 8.7 [0.0]  1 (4.0) / 8.5 [11.7] |
| Infections and infestations  Catheter site infection  Device related infection  Gastroenteritis  Influenza  Nasopharyngitis  Peritonitis  Pneumonia  Upper respiratory tract infection | 0 (0.0) / 9.7 [0.0]  2 (9.5) / 9.0 [22.3]  1 (4.8) / 9.2 [10.8]  1 (4.8) / 9.5 [10.5]  1 (4.8) / 9.1 [11.0]  0 (0.0) / 9.7 [0.0]  0 (0.0) / 9.7 [0.0]  4 (19.0) / 8.4 [47.5] | 2 (9.1) / 7.9 [25.3]  2 (9.1) / 8.5 [23.6]  0 (0.0) / 8.7 [0.0]  3 (13.6) / 7.8 [38.3]  2 (9.1) / 8.2 [24.3]  1 (4.5) / 8.4 [11.8]  0 (0.0) / 8.7 [0.0]  0 (0.0) / 8.7 [0.0] | 0 (0.0) / 10.9 [0.0]  0 (0.0) / 10.9 [0.0]  0 (0.0) / 10.9 [0.0]  0 (0.0) / 10.9 [0.0]  2 (6.7) / 10.2 [19.7]  0 (0.0) / 10.9 [0.0]  0 (0.0) / 10.9 [0.0]  0 (0.0) / 10.9 [0.0] | 0 (0.0) / 8.7 [0.0]  1 (4.0) / 8.3 [12.0]  2 (8.0) / 8.3 [24.2]  0 (0. 0) / 8.7 [0.0]  2 (8.0) / 8.4 [23.8]  2 (8.0) / 8.1 [24.7]  2 (8.0) / 8.4 [23.8]  0 (0.0) / 8.7 [0.0] |
| Injury, poisoning and procedural complications  Arteriovenous fistula site complication  Procedural pain | 3 (14.3) / 9.3 [32.4]  0 (0.0) / 9.7 [0.0] | 0 (0.0) / 8.7 [0.0]  0 (0.0) / 8.7 [0.0] | 1 (3.3) / 10.7 [9.4]  2 (6.7) / 10.4 [19.2] | 1 (4.0) / 8.5 [11.8]  0 (0.0) / 8.7 [0.0] |
| Investigations  Weight increased | 0 (0.0) / 9.7 [0.0] | 0 (0.0) / 8.7 [0.0] | 2 (6.7) / 10.5 [19.0] | 0 (0.0) / 8.7 [0.0] |
| Metabolism and nutrition disorders  Dehydration  Hyperkalemia  Hypocalcemia  Vitamin D deficiency | 2 (9.5) / 9.0 [22.1]  3 (14.3) / 8.8 [34.0]  4 (19.0) / 8.5 [47.1]  2 (9.5) / 9.3 [21.5] | 0 (0.0) / 8.7 [0.0]  1 (4.5) / 8.6 [11.6]  5 (22.7) / 7.4 [67.6]  0 (0.0) / 8.7 [0.0] | 0 (0.0) / 10.9 [0.0]  0 (0.0) / 10.9 [0.0]  2 (6.7) / 10.5 [19.0]  0 (0.0) / 10.9 [0.0] | 0 (0.0) / 8.7 [0.0]  0 (0.0) / 8.7 [0.0]  6 (24.0) / 7.6 [79.0]  0 (0.0) / 8.7 [0.0] |
| Musculoskeletal and connective tissue disorders  Back pain  Muscle spasms  Musculoskeletal stiffness  Myalgia  Pain in extremity | 2 (9.5) / 9.2 [21.7]  1 (4.8) / 9.2 [10.9]  0 (0.0) / 9.7 [0.0]  1 (4.8) / 9.4 [10.6]  0 (0.0) / 9.7 [0.0] | 0 (0.0) / 8.7 [0.0]  3 (13.6) / 8.1 [37.0]  2 (9.1) / 8.2 [24.4]  3 (13.6) / 8.0 [37.3]  1 (4.5) / 8.4 [11.9] | 0 (0.0) / 10.9 [0.0]  2 (6.7) / 10.3 [19.3]  0 (0.0) / 10.9 [0.0]  1 (3.3) / 10.8 [9.2]  2 (6.7) / 10.2 [19.6] | 0 (0.0) / 8.7 [0.0]  3 (12.0) / 7.9 [38.0]  0 (0.0) / 8.7 [0.0]  0 (0.0) / 8.7 [0.0]  0 (0.0) / 8.7 [0.0] |
| Nervous system disorders  Dizziness  Headache | 0 (0.0) / 9.7 [0.0]  2 (9.5) / 9.1 [22.1] | 2 (9.1) / 8.1 [24.6]  3 (13.6) / 8.0 [37.5] | 0 (0.0) / 10.9 [0.0]  4 (13.3) / 9.9 [40.2] | 0 (0.0) / 8.7 [0.0]  1 (4.0) / 8.4 [12.0] |
| Psychiatric disorders  Anxiety | 0 (0.0) / 9.7 [0.0] | 2 (9.1) / 8.2 [24.3] | 0 (0.0) / 10.9 [0.0] | 0 (0.0) / 8.7 [0.0] |
| Respiratory, thoracic and mediastinal disorders  Cough  Nasal congestion  Oropharyngeal pain | 3 (14.3) / 9.0 [33.4]  3 (14.3) / 9.0 [33.3]  2 (9.5) / 9.4 [21.2] | 1 (4.5) / 8.2 [12.2]  0 (0.0) / 8.7 [0.0]  0 (0.0) / 8.7 [0.0] | 1 (3.3) / 10.4 [9.6]  0 (0.0) / 10.9 [0.0]  0 (0.0) / 10.9 [0.0] | 0 (0.0) / 8.7 [0.0]  0 (0.0) / 8.7 [0.0]  0 (0.0) / 8.7 [0.0] |
| Vascular disorders  Hypertension  Hypotension | 5 (23.8) / 7.5 [66.4]  1 (4.8) / 9.4 [10.6] | 3 (13.6) / 8.3 [36.1]  2 (9.1) / 8.4 [23.9] | 1 (3.3) / 10.5 [9.6]  0 (0.0) / 10.9 [0.0] | 1 (4.0) / 8.6 [11.7]  0 (0.0) / 8.7 [0.0] |

Coded using MedDRA version v19.0.
N, number of subjects in the analysis set; n, number of subjects reporting at least 1 occurrence of an adverse event. Percentages are based on N.

^a^Study 20070208 control = placebo; Study 20130356 control = standard of care.

e, sum across all subjects, the total time to first event or total safety follow-up if no event (years); r, Safety follow-up adjusted subject rate per 100 subject years (n/e*100).

**Table S5.** Concomitant phosphate binder and vitamin D use

|  | **20070208** | | **20130356** | | **20110100** | | **20140159** |
| --- | --- | --- | --- | --- | --- | --- | --- |
|  | **Cinacalcet**  **(n = 22)** | **PBO**  **(n = 21)** | **Cinacalcet  + SOC**  **(n = 27)** | **SOC**  **(n = 28)** | **Cohort 1**  **(n = 8)^a^** | **Cohort 2**  **(n = 10)** | **Cinacalcet**  **(n = 13)^b^** |
| **Phosphate binder use at baseline^c^, n (%)** | 21 (95.5) | 20 (95.2) | 15 (55.6) | 18 (64.3) | 5 (62.5) | 8 (80.0) | 8 (61.5) |
| Calcium-containing | 15 (68.2) | 15 (71.4) | 9 (33.3) | 8 (28.6) | 1 (12.5) | 4 (40.0) | 3 (23.1) |
| Magnesium-containing | 0 (0.0) | 0 (0.0) | 0 (0.0) | 0 (0.0) | 0 (0.0) | 0 (0.0) | 0 (0.0) |
| Aluminum-containing | 0 (0.0) | 0 (0.0) | 0 (0.0) | 0 (0.0) | 0 (0.0) | 0 (0.0) | 0 (0.0) |
| Sevelamer HCl | 9 (40.9) | 9 (42.9) | 4 (14.8) | 1 (3.6) | 0 (0.0) | 1 (10.0) | 2 (15.4) |
| Lanthanum carbonate | 2 (9.1) | 0 (0.0) | 0 (0.0) | 0 (0.0) | 0 (0.0) | 0 (0.0) | 0 (0.0) |
| Sevelamer Carbonate | 3 (13.6) | 6 (28.6) | 5 (18.5) | 9 (32.1) | 4 (50.0) | 4 (40.0) | 5 (38.5) |
| Other | 0 (0.0) | 0 (0.0) | n/a | n/a | 0 (0.0) | 1 (10.0) | n/a |
| **Initiated phosphate binder use post-baseline, n (%)** | 1 (4.5) | 1 (4.8) | 0 (0.0)^d^ | 4 (13.3)^e^ | 1 (14.3)^f^ | 0 (0.0) | 0 (0.0) |
| **Vitamin D sterol use at baseline** | 22 (100.0) | 19 (90.5) | 19 (70.4) | 22 (78.6) | 7 (87.5) | 9 (90.0) | 10 (76.9) |
| IV Calcitriol | 1 (4.5) | 2 (9.5) | 0 (0.0) | 1 (3.6) | 1 (12.5) | 0 (0.0) | 0 (0.0) |
| Oral Calcitriol | 7 (31.8) | 3 (14.3) | 4 (14.8) | 6 (21.4) | 1 (12.5) | 5 (50.0) | 0 (0.0) |
| IV Paricalcitol | 5 (22.7) | 8 (38.1) | 9 (33.3) | 1 (3.6) | 2 (25.0) | 1 (10.0) | 1 (7.7) |
| Oral Paricalcitol | 4 (18.2) | 1 (4.8) | 0 (0.0) | 0 (0.0) | 0 (0.0) | 0 (0.0) | 2 (15.4) |
| Oral Alfacalcidol | 8 (36.4) | 5 (23.8) | 7 (25.9) | 12 (42.9) | 3 (37.5) | 2 (20.0) | 7 (53.8) |
| **Initiated vitamin D sterol use post-baseline, n (%)** | 1 (4.5) | 2 (9.5) | 2 (8.0)^d^ | 3 (10.0)^e^ | 1 (14.3)^f^ | 1 (10.0) | 1 (7.7) |
| **Nutritional vitamin D use at baseline, n (%)** | 9 (40.9) | 10 (47.6) | 7 (25.9) | 13 (46.4) | 5 (62.5) | 5 (50.0) | 5 (38.5) |
| Cholecalciferol | 7 (31.8) | 6 (28.6) | 4 (14.8) | 11 (39.3) | 4 (50.0) | 3 (30.0) | 4 (30.8) |
| Ergocalciferol | 2 (9.1) | 4 (19.0) | 3 (11.1) | 2 (7.1) | 1 (12.5) | 2 (20.0) | 1 (7.7) |
| **Initiated nutritional vitamin D use post-baseline, n (%)** | 1 (4.5) | 4 (19.0) | 2 (8.0)^d^ | 2 (6.7)^e^ | 0 (0.0) | 1 (10.0) | 0 (0.0) |

^a^Based on enrolled patients (n = 8);^b^Only 13 patients receiving SOC in Study 20130356 that received cinacalcet in Study 20140159 were considered.^c^Subjects could use multiple types of phosphate binder; the subcategories are not mutually exclusive; ^d^Based on safety analysis set (n = 25); ^e^Based on safety analysis set (n = 30); ^f^Based on safety analysis set (n = 7)

IV, intravenous; PBO, placebo; SOC, standard of care
