# Supplementary material for: Cinacalcet studies in pediatric subjects with secondary hyperparathyroidism receiving dialysis
Source: Pediatr Nephrol. 2020 May 4;35(9):1679–97. doi: 10.1007/s00467-020-04516-4 (PMC7385021; doi:10.1007/s00467-020-04516-4)
Supplement: Supplementary file 2 — (PPTX 63 kb) [file 467_2020_4516_MOESM2_ESM.pptx]

## Slide 1
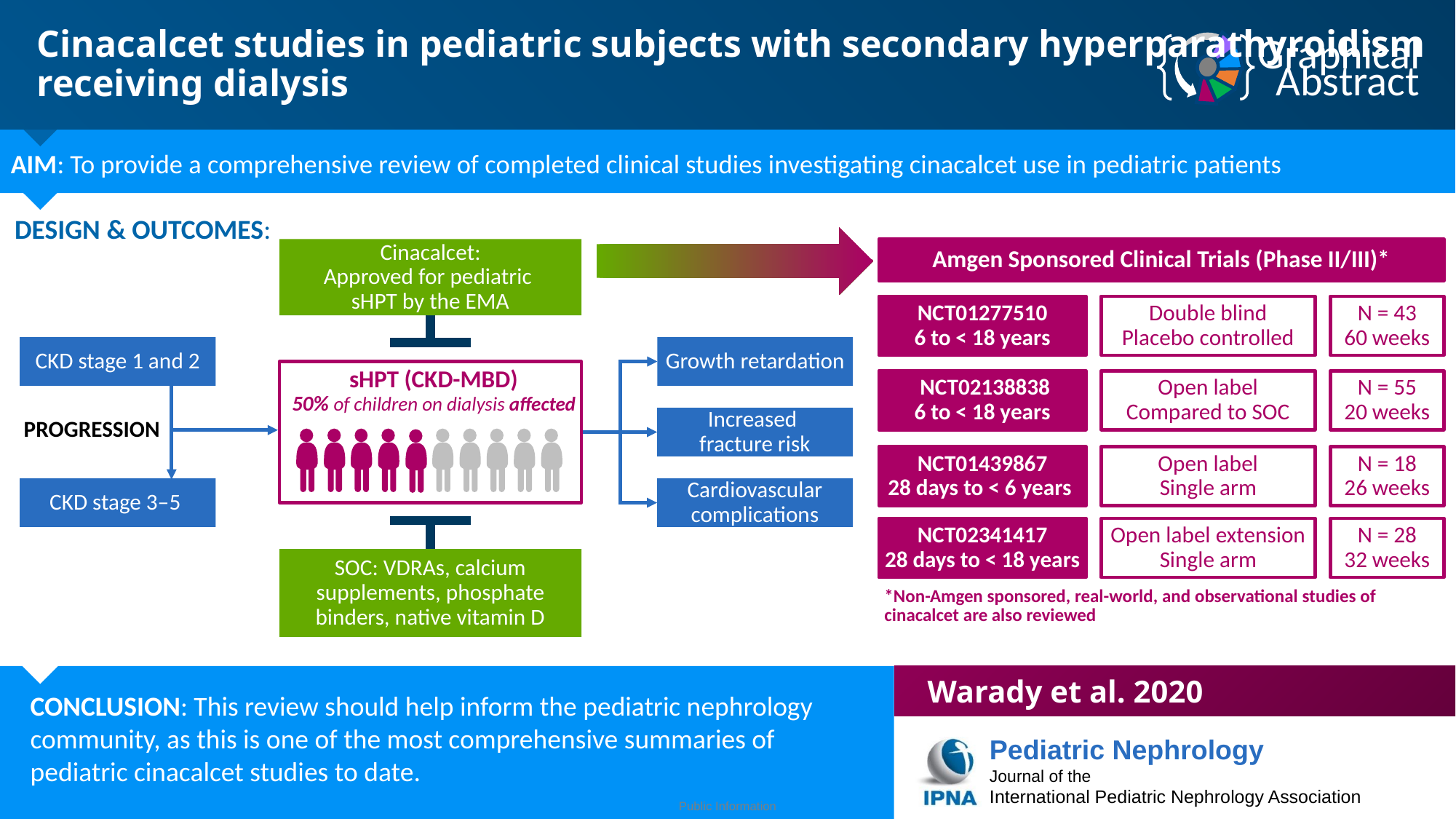

Cinacalcet studies in pediatric subjects with secondary hyperparathyroidism
receiving dialysis
Cinacalcet:
Approved for pediatric
sHPT by the EMA
AIM: To provide a comprehensive review of completed clinical studies investigating cinacalcet use in pediatric patients
DESIGN & OUTCOMES:
Amgen Sponsored Clinical Trials (Phase II/III)*
NCT01277510
6 to < 18 years
Double blind
Placebo controlled
N = 43
60 weeks
 NCT02138838
6 to < 18 years
Open label
Compared to SOC
N = 55
20 weeks
NCT01439867
28 days to < 6 years
Open label
Single arm
N = 18
26 weeks
NCT02341417
28 days to < 18 years
Open label extension
Single arm
N = 28
32 weeks
*Non-Amgen sponsored, real-world, and observational studies of cinacalcet are also reviewed
CKD stage 1 and 2
Growth retardation
Increased
fracture risk
sHPT (CKD-MBD)
50% of children on dialysis affected
CKD stage 3‒5
Cardiovascular complications
PROGRESSION
SOC: VDRAs, calcium supplements, phosphate binders, native vitamin D
Warady et al. 2020
CONCLUSION: This review should help inform the pediatric nephrology community, as this is one of the most comprehensive summaries of pediatric cinacalcet studies to date.
Public Information
